# Quantitative Analysis of Impurities in Unfractionated Heparin of Bovine Origin

**DOI:** 10.3389/fmed.2019.00315

**Published:** 2020-01-10

**Authors:** Wesley E. Workman, Kevin L. Carrick

**Affiliations:** Global Biologics, United States Pharmacopeia, Rockville, MD, United States

**Keywords:** heparin, bovine, porcine, dermatan, galactosamine, OSCS

## Abstract

The USP heparin sodium monograph lists impurities with specifications developed for porcine derived products. Most of these impurities are of biological origin and are present in porcine intestinal mucosa, the tissue source used in the production of porcine heparin. One of the specified impurities, oversulfated chondroitin sulfate (OSCS), has been introduced in the monograph to detect intended adulteration of heparin products with this impurity. The evaluation of bovine intestinal heparin as an alternative source of pharmaceutical heparin included an evaluation of bovine heparin with the current USP heparin sodium monograph methods. This evaluation included a comparison of impurity quantities observed in multiple bovine intestinal heparin samples against the specifications found in the USP heparin sodium monograph. The impurities investigated in this study were protein, galactosamine, nucleotidic impurities, and OSCS. Bovine intestinal heparin met the requirements in the tests for protein, galactosamine, and nucleotidic impurities. A potential issue was observed with the strong anion exchange high performance liquid chromatography (SAX-HPLC) used to analyze for the presence of OSCS. While the OSCS was well-resolved from the bovine heparin peak, the resolution of dermatan sulfate from heparin did not consistently meet system suitability requirements in the current USP Heparin sodium monograph. The overall levels of impurities observed in bovine intestinal mucosal heparin were comparable to those observed in porcine intestinal mucosal heparin. Bovine intestinal mucosal heparin can be produced with acceptable impurity levels that align with these important quality attributes found in porcine heparin.

## Introduction

Heparin is a valuable animal-derived pharmaceutical that the medical community depends on to control blood coagulation in a number of patient applications. Likewise, the research community also relies on heparin as a critical reagent in many research applications. Therefore, a consistent supply of high quality heparin is needed for various purposes that contribute to immediate and long term medical needs of patients now and in the future. Heparin is most often obtained by extraction from porcine intestinal mucosa as a by-product of the pork industry. The need for porcine mucosa as the starting raw material for heparin production can lead to pressures on the supply chain that are not often not seen with pharmaceutical products produced through chemical processes. Those pressures can include disease, agricultural economics, and regional differences with regard to animal-derived protein sources (1). These pressures led to an evaluation of bovine-derived heparin as an alternative to porcine-derived heparin for pharmaceutical use.

In the past, bovine lung has been used as a starting raw material for heparin production. Concerns with transmissible spongiform encephalopathy (TSE), or more specifically bovine spongiform encephalopathy (BSE), led to bovine-derived heparin falling out of favor for pharmaceutical use. Prions, the causative agents that can result in the BSE associated neurodegenerative disorders, are particularly resistant to inactivation and those concerns were not associated with porcine-derived heparin. As agricultural practices advanced, risk assessments indicated that bovine-derived heparin may be an acceptable alternative to porcine derived heparin (2).

Naturally occurring impurities found in porcine-derived heparin are defined by the USP heparin sodium monograph (3) with specific analytical tests and limits. Those impurities include galactosamine impurity, nucleotidic impurities, and protein impurities. Additionally, an analytical test for the absence of OSCS is also included in the impurities section of the USP heparin sodium monograph. The current USP heparin sodium monograph is the result of the evolution of the monograph over three “Stages” from 2008 through 2014 with the impurity limits being tightened during later Stages (4). The tightening of the specification limits in the porcine heparin monograph reflects what is expected from a well-developed heparin purification process and resulted in high quality consistent heparin sodium active pharmaceutical ingredient (API) being produced for use in the USA. As stated previously, bovine-derived heparin was obtained from bovine lung tissue in the past, but current economics have led to the majority of bovine-derived heparin being produced from bovine intestinal mucosa. To evaluate whether the USP heparin sodium monograph analytical tests and limits for impurities could be applied to bovine intestinal heparin sodium API, a study involving multiple laboratories was performed with the results provided in this report.

## Materials and Methods

Analytical methods and acceptance criteria for porcine intestinal heparin are described in the USP monograph (3). The suitability of those analytical methods and acceptance criteria in the testing of bovine heparin were evaluated with 13 samples of bovine intestinal heparin from two manufacturers, nine lots manufacturer 1 and four lots manufacturer 2. One sample of porcine heparin sodium was included as a control. The test and control samples were analyzed for impurities [i.e., protein, galactosamine, nucleotidic impurities, and oversulfated chondroitin sulfate (OSCS)] in a study by three independent laboratories. Specific details on performance of the analytical methods, including source of reference standards, chromatography columns, and system suitability criteria, can be found in the USP heparin sodium monograph (3).

### Protein Impurities

Protein impurities were determined using a modified Lowry protein determination method. The standard curve for protein determination was prepared using bovine serum albumin. An optional sample preparation to remove substances that interfered with the protein determination was performed as needed. This optional step was referred to as the Interfering Substances Treatment (IST). The acceptance criterion was a protein content in the heparin sample that did not exceed 0.1% (w/w).

### Limit of Galactosamine in Total Hexosamine

The limit of galactosamine in total hexosamine was determined by hydrolyzing the sample with 5 N hydrochloric acid. The amount of galactosamine and glucosamine in the sample after hydrolysis was determined by high pressure ion chromatography (HPIC). The acceptance criterion was a galactosamine peak area that did not exceed 1% of the total hexosamine peak area.

### Nucleotidic Impurities

The nucleotidic impurities were determined by first digesting nucleic acids in the heparin sample with a combination of enzymes to generate free nucleosides. The resulting nucleosides were separated by high pressure liquid chromatography (HPLC) and the peak areas of the nucleosides were determined. The percentage of each nucleoside in the heparin sample was determined by comparing its peak area to that of an adenosine reference standard. The acceptance criterion was a nucleotidic impurity content in the heparin sample that did not exceed 0.1% (w/w).

### Absence of Oversulfated Chondroitin Sulfate

The absence of OSCS was determined using strong anion exchange HPLC (SAX-HPLC). The applicability of the SAX-HPLC technique to bovine heparin was evaluated based on the ability to separate and quantitate heparin, dermatan sulfate, and OSCS. The acceptance criterion was no peak corresponding to OSCS should be detected.

## Results

Heparin is extracted from animal tissues using protease (5). This extraction step can result in residual proteins being present in the heparin that are removed through the purification steps associated with production of the heparin API. Low levels of protein impurity [i.e., not more than the acceptance criterion of 0.1% (w/w)] demonstrate the efficiency of the purification process with respect to this quality attribute. The modified Lowry protein determination was used to quantitate the protein content of the heparin samples. The IST is an optional sample preparation step described in the USP heparin sodium monograph (3). Some laboratories may find that the heparin API samples contain interfering substances that give a non-protein related response in the modified Lowry protein determination. This non-protein related response may cause the result to falsely exceed the 0.1% (w/w) protein content acceptance criterion. Therefore, the option is given to use an IST. The IST involves, (1) add deoxycholate to the sample, (2) precipitate the protein in the sample with trichloroacetic acid, (3) centrifuge the sample to pellet the protein, (4) pour off the supernatant that contains the potential interfering substances, and (5) dissolve the protein pellet in a small volume of one of the modified Lowry protein determination reagents. Some laboratories choose to use an IST with every sample for efficiency, rather than repeating testing on a failing sample with an IST to determine if it passes the protein content acceptance criterion. Table 1 gives the results of the protein determination for each sample by the three participating laboratories, two of which used the IST as indicated in Table 1. The results obtained from the two laboratories using the IST were lower than the results from the laboratory not using the IST, demonstrating the value in using the IST. All results obtained with bovine intestinal heparin met the acceptance criterion for protein determination of not more than 0.1% (w/w), suggesting that bovine intestinal heparin could be manufactured to meet the protein impurity quality attribute used to assess porcine intestinal heparin.

**Table 1:** Protein content determination of bovine intestinal heparin.

**Sample number**	**Percent protein content (% w/w)**		
	**Laboratory 1**	Laboratory 2^*^	Laboratory 3^*^
Porcine heparin control	0.18%	0.08%	0.10%
K-1	0.10%	0.03%	0.03%
L-1	0.08%	0.02%	0.04%
M-1	0.08%	0.01%	0.03%
N-1	0.08%	0.02%	NA
O-1	0.08%	0.02%	NA
O-2	0.10%	0.04%	0.04%
P-1	0.08%	0.02%	NA
P-2	0.07%	0.03%	0.03%
Q-1	0.07%	0.02%	NA
Q-2	0.06%	0.03%	0.03%
R-1	0.07%	0.01%	NA
R-2	0.05%	0.02%	NA
S-1	0.06%	0.01%	NA

* *Laboratories used IST for sample preparation prior to analysis. NA indicates the sample results were not available*.

Heparin, dermatan sulfate, and chondroitin sulfate are all members of a molecular species known collectively as glycosaminoglycans (GAGs). All are polysaccharides with heparin containing glucosamine whereas dermatan sulfate and chondroitin sulfate contain galactosamine. Dermatan sulfate differs from chondroitin sulfate in that dermatan sulfate also contains iduronic acid, whereas chondroitin sulfate contains glucuronic acid. When heparin is extracted from animal tissues, other GAGs are among the naturally occurring molecules that may also be extracted. The commercial heparin purification process is intended to remove GAGs other than heparin. Therefore, the determination of the percentage of galactosamine in total hexosamine as described in the USP heparin sodium monograph will determine the content of GAG impurities in the heparin product and assess the efficiency of the heparin purification process. An acceptance criterion of not more than 1% galactosamine in total hexosamine is the limit used in the USP heparin sodium monograph. Table 2 shows the results obtained by two of the participating laboratories.

**Table 2:** Galactosamine content determination of bovine intestinal heparin.

**Sample number**	**Percent galactosamine in total hexosamine**	
	**Laboratory 1**	Laboratory 2^*^
Porcine heparin control	0.1%	0.37%
K-1	<0.05%	ND
L-1	<0.05%	ND
M-1	<0.05%	ND
N-1	<0.05%	ND
O-1	<0.05%	ND
O-2	0.1%	0.08%
P-1	<0.05%	ND
P-2	<0.05%	ND
Q-1	<0.05%	ND
Q-2	<0.05%	<0.05%
R-1	<0.05%	ND
R-2	<0.05%	ND
S-1	0.1%	ND

* *ND indicates that no galactosamine was detected*.

During heparin extraction from raw tissue, nucleotidic impurities such as deoxyribonucleic acid (DNA) and ribonucleic acid (RNA) can be extracted along with the heparin (5). Heparin and nucleic acids share common chemistry composition in that they are polymers with a significant negative charge and both are purified by ethanol precipitation. Removal of nucleic acids during the heparin purification process is one of the quality attributes that indicates if a heparin purification process is performing efficiently and adequately removing impurities. Table 3 shows the results obtained from one laboratory for nucleotidic impurity content in bovine intestinal heparin.

**Table 3:** Nucleotidic impurity content determination of bovine heparin.

**Sample number**	Percent nucleotidic impurity^*^
Porcine heparin control	<0.05%
K-1	ND
L-1	ND
M-1	<0.05%
N-1	ND
O-1	ND
O-2	ND
P-1	<0.05%
P-2	ND
Q-1	ND
Q-2	ND
R-1	ND
R-2	ND
S-1	ND

* *ND indicates that no nucleotidic impurities were detected in the sample. The Quantitation Limit for the Nucleotidic Impurity Assay is 0.05%*.

**Figure 1:**
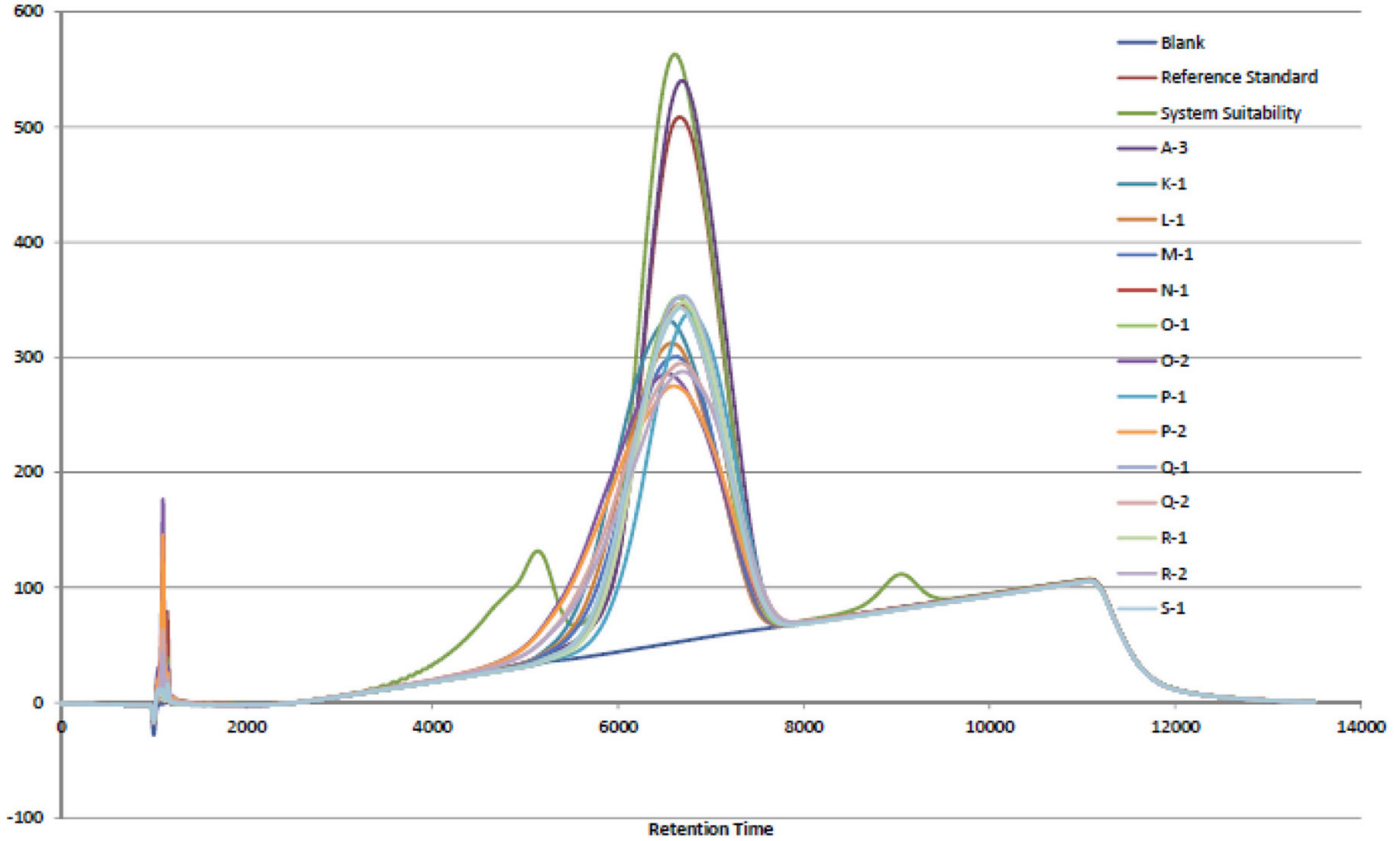
SAX-HPLC chromatogram overlay of porcine intestinal heparin (Sample A-3) and bovine intestinal heparin (K-1, L-1, M-1, N-1, O-1, O-2, P-1, P-2, Q-1, Q-2, R-1, R-2, S-1). Blank, reference standard, and system suitability control, containing dermatan sulfate (eluting before the heparin peak) and OSCS (eluting after the heparin peak), are shown.

In 2007 and 2008, an adulterant was found in the commercial porcine heparin supply that was identified as OSCS. Lack of specific tests for heparin at the time may have led to the opportunity for this adulteration. The USP heparin sodium monograph (3) has modified sections for Identification and Impurities with analytical methods and limits that support the Absence of OSCS acceptance criterion requirement. Those analytical methods are NMR and SAX-HPLC. The USP chose to use the SAX-HPLC analytical method due to (1) resolution of the analyte species, (2) mobile phase suitability, and (3) column matrix suitability (4). The results shown in Figure 1 indicate that the retention time for the bovine intestinal heparin was equivalent to the porcine intestinal heparin and OSCS was fully resolved in the chromatogram. It was noted that dermatan sulfate was not fully resolved in the SAX-HPLC chromatogram (Figure 1) and may need to be further evaluated if the goal is to apply the USP heparin sodium monograph analytical methods to bovine intestinal heparin in the future.

## Discussion

Bovine intestinal heparin API can be manufactured to meet the same quality criteria as porcine intestinal heparin API with regards to the detected impurities. The analytical methods described in the USP heparin sodium monograph can be used to assess the quality of bovine heparin sodium. Further development may be needed to address the dermatan sulfate resolution in the SAX-HPLC assay for the USP heparin sodium monograph to be used with bovine heparin API, however the amount of dermatan sulfate impurity in the bovine heparin API can be controlled by the galactosamine assay and specifications.

## Data Availability Statement

The datasets for this manuscript are not publicly available because the data is contained within the United States Pharmacopeial archives, but is being published in its entirety in the article. Requests to access the datasets should be directed to Kevin Carrick, KLC@usp.org.

## Author Contributions

WW provided oversight of the analytical studies. WW and KC shared responsibilities for the preparation of the manuscript.

### Conflict of Interest

WW is an employee of Pfizer, Inc., a manufacturer of heparin products. The remaining author declares that the research was conducted in the absence of any commercial or financial relationships that could be construed as a potential conflict of interest.
